# Antibacterial activity of oregano essential oils against *Streptococcus mutans* in vitro and analysis of active components

**DOI:** 10.1186/s12906-023-03890-4

**Published:** 2023-02-21

**Authors:** Yue Yuan, Jinlong Sun, Yang Song, Rifat Nowshin Raka, Jie Xiang, Hua Wu, Junsong Xiao, Jianming Jin, XiuLi Hui

**Affiliations:** 1 Beijing Technology and Business University, Beijing, 100048 China; 2 Department of Stomatology, Sixth Medical Center of PLA General Hospital, Beijing, 100048 China

**Keywords:** Oregano essential oil, GC–MS, *Streptococcus mutans*, Dental caries

## Abstract

**Background:**

*Streptococcus mutans* (*S. mutans*) is considered the most relevant bacteria during the transition of the non-pathogenic commensal oral microbial community to plaque biofilms that promote the development of dental caries. Oregano (*Origanum vulgare* L*.*), is a universally natural flavoring and its essential oil has been demonstrated to have good antibacterial effects. However, the specific antibacterial mechanism of oregano essential oil (OEO) against *S. mutans* is still not completely understood.

**Methods:**

In this work, the composition of two different OEOs was determined by GC‒MS. Disk-diffusion method, minimum inhibitory concentration (MIC) and minimum bactericidal concentration (MBC) were determined to assess their antimicrobial effect on *S. mutans*. The inhibition of acid production, hydrophobicity, biofilm formation and real-time PCR for *gtfB*/*C*/*D*, *spaP*, *gbpB*, *vicR*, *relA* and *brpA* mRNA expression by *S. mutans* were assessed to preliminarily investigate the mechanisms of action. Molecular docking was performed to simulate the interactions with the virulence proteins and active constituents. MTT test using immortalized human keratinocytes cells was also performed to investigate cytotoxicity.

**Results:**

Compared with the positive drug Penicillin /streptomycin 100X (DIZ: 34.13 ± 0.85 mm, MIC: 0.78125 μL/mL, MBC: 6.25 μL/mL), the essential oils of *Origanum vulgare* L*.* (DIZ: 80 mm, MIC: 0.625μL/mL, MBC:2.5μL/mL) and *Origanum heracleoticum* L*.* (DIZ: 39.67 ± 0.81 mm, MIC: 0.625μL/mL, MBC: 1.25μL/mL) could also exhibit similar effects to inhibit the acid production and reduce the hydrophobicity and biofilm formation of *S. mutans* at 1/2-1MIC concentration. And gene expression of *gtfB/C/D*, *spaP*, *gbpB*, *vicR* and *relA* were found to be downregulated. Due to the composition of essential oils from different sources being highly variable, through effective network pharmacology analysis, we found that OEOs contained many effective compounds, like carvacrol and its biosynthetic precursors *γ*-terpinene and *p*-cymene, which may directly target several virulence proteins of *S. mutans***.** Besides, no toxic effect was instigated by OEOs at 0.1 μL/mL in the immortalized human keratinocytes cells.

**Conclusion:**

The integrated analysis in the present study suggested that OEO might be a potential antibacterial agent for the prevention of dental caries.

**Supplementary Information:**

The online version contains supplementary material available at 10.1186/s12906-023-03890-4.

## Introduction

The public health challenges posed by dental caries are numerous. The burden of disease study revealed in a systematic analysis that dental caries is one of the most prevalent chronic diseases globally, affecting around 2.4 billion people [1]. Due to the substantial decline in quality of life and expensive out-of-pocket payments, an increased interest is driving the transition from surgical management to prevention of caries. In our complex oral microbiota, there is a clear association between abnormal levels of *Streptococcus mutans* (*S. mutans*) and caries development [2]. This means that one effective and feasible approach to rebuilding a healthy bacterial community is to reduce and balance the prevalence of *S. mutans*. The current established caries-preventive measures include maintaining oral hygiene, regular dental checkups, reducing sugar intake (like using commercially available sugar substitutes), increasing fluoride availability (supplement in water, toothpaste, mouth rinse, or varnish), and placing of fissure sealants [3, 4]. Despite these public measures, dental caries is still prevalent in the population due to the existence of different daily habits, differences in microbiome strains and virulence, oral health literacy, and socioeconomic vulnerability. Therefore, more safe and effective preventive strategies for dental caries are still needed, especially in populations with difficulties in regular follow-up or in the practice of mechanical oral hygiene.

Plants are a good source of natural products and are extensively used in traditional medicine, food, and cosmetics [5–7]. *Origanum vulgare* L., commonly known as oregano, is a perennial plant belonging to the Lamiaceae family native to the Mediterranean region and western Eurasia. It is an herb traditionally used for flavoring the cuisine [8]. The essential oils extracted from its leaves exhibited numerous useful biological activities in oral care and dental practice. For example, recent studies have found that oregano essential oil (OEO) has antibacterial effects against clinically isolated oral *Candida*, and thus may hold great potential for the prevention and treatment of *Candida*-associated denture stomatitis [9]. Toothpaste containing OEO was able to completely disrupt biofilms produced by cariogenic *Streptococcus mutans* (*S. mutans*) [10]. Mohamed Saeed et al. [11] used OEO mouthwash, chlorhexidine, and a placebo in 54 patients with halitosis. The results of the organoleptic method and the BANA test of the tongue showed that OEO mouthwash was similar to chlorhexidine in reducing oral malodor, possibly due to its potent antibacterial properties. The main component of OEO, carvacrol, as a substitute for carbolic acid and creosote, has also been used for sensitive dentine, odontalgia, alveolar abscess, and root canal and periapical infections treatment. Moreover, carvacrol also possesses antioxidant, anti-inflammatory, hepatoprotective, antispasmodic, and antitumor activities [12].

However, so far, its mechanism of action of OEO to prevent dental caries by inhibiting oral bacteria *S. mutans* remains unclear. With this backdrop, we sought to investigate the effects of the OEO on the *S. mutans* and to evaluate the safety/toxicity of this OEO when in contact with human keratinocytes. Moreover, we analyzed the interaction of key ingredients, including carvacrol and its biosynthetic precursors *γ*-terpinene and *p*-cymene, with *S. mutans* target virulence proteins via network pharmacology-based prediction.

## Materials and methods

### Chemicals and reagents

Essential oil of *Origanum vulgare* L*.* (OEO1, origin: Bosnia) and *Origanum heracleoticum* L*.* (OEO2, origin: France) were purchased from Poli Aromatic Pharmaceutical Technology Co., Ltd (Shanghai, China). Dichloromethane (≥ 99.9%, HPLC grade) was obtained from Innochem Science & Technology Co., Ltd (Beijing, China). K_2_HPO_4_‧3H_2_O, KH_2_PO_4_, MgSO_4_, urea, hexadecane and glucose were all purchased from Energy Chemical Co., Ltd (Shanghai, China). Tween 80 was provided by Shandong Usolf Chemical Industry Co., Ltd (Shandong, China). Sucrose was provided by Tianjin Fuchen Chemical Reagent Factory (Tianjin, China). Crystal violet and safranin reagents were purchased from Beijing Solarbio Technology Co., Ltd (Beijing, China). 3-(4,5-Dimethylthiazol-2-yl)-2,5-diphenyltetrazolium (MTT) and fetal bovine serum (FBS) were obtained from Beyotime Biotechnology Co., Ltd (Shandong, China). Penicillin /streptomycin 100X (P/S, 10,000 units penicillin and 10 mg streptomycin/mL) and glutamine were obtained from Zhongsheng Aobang Biotechnology Co., LTD (Beijing, China). Dulbecco’s Modified Eagle’s Medium (DMEM) and phosphate buffered solution (PBS) were purchased from Hyclone Co., Ltd (USA).

### Microorganisms and culture conditions

*Streptococcus mutans* ATCC 700,610 (*S. mutans*) was purchased from China General Microbiological Culture Collection Center (Beijing, China). In Brain Heart Infusion (BHI) broth (Oxoid Ltd, England), the strain was cultured for 24 h at 37 °C. The bacterial concentration was diluted to 1 × 10^5^ CFU/ml for further studies, if not otherwise stated.

### GC–MS analysis

The essential oils were analyzed using an Agilent 7890B/5977A gas chromatograph (Agilent Technologies) equipped with a 60 m × 0.25 mm × 0.25 μm DB-WAX column. The mass-selective detector operated in scan range from m/z 30 to m/z 550 at 70 eV. Initially, the oven temperature was 40 °C, and then raised in stepwise manner to 50 °C, 92 °C, 120 °C and 230 °C at a rate of 5 °C/min, 6 °C/min, 3 °C/min and 5 °C/min, respectively. The carrier gas was helium, and the flow rate was 1.2 mL/min. The sample diluted with dichloromethane was injected into the gas chromatograph with a split ratio of 33.3. By comparing with the National Institute of Standards and Technology (NIST) MS spectral database (version 2017), the sample constituents were identified [13].

### Preparation of samples

The OEOs were dissolved in Tween 80 (0.1% in BHI broth) to a stock concentration of 20 μL/mL. Then serial twofold dilutions were performed to obtain serial OEOs and P/S concentrations ranging from 0.625 to 20 μL/mL and 0.390625 to 50 μL/mL for the evaluation [14]. And it was required for sample preparation in minimum inhibitory concentration, minimum inhibitory concentration, acid production, hydrophobicity, biofilm formation, and quantitative real-time polymerase chain reaction experiments.

### Evaluation of antibacterial activities of OEOs

#### Antibacterial activities of OEOs

Disk diffusion method was employed for antimicrobial susceptibility testing [15]. Briefly, 100 µL bacterial suspension was spread evenly on BHI agar plates. A 6 mm sterile filter paper disc impregnated with 10 µL of the pure essential oil (or P/S) was placed at the center of the plate. After incubating at 37 °C for 24 h under anaerobic conditions, the diameters of the zones in millimeters on the agar plates were measured. P/S and BHI broth were used in parallel experiments as the positive and blank controls respectively.

#### Determination of MIC (Minimum inhibitory concentration)/MBC (Minimum inhibitory concentration)

Minimum inhibitory concentration (MIC) and minimum bactericide concentration (MBC) were performed as described with a few modifications [16]. Briefly, serial two-fold dilutions of samples (0.625–20 μL/mL) and P/S (0.390625–50 μL/mL) were incubated with bacteria for 24 h at 37 ℃. Bacterial suspension containing 0.1% (v/v) Tween 80 served as negative control. MIC was defined as the lowest concentration of essential oils inhibiting visible bacterial growth. MBC was determined by employing the tubes without visual bacteria growth and then sub culturing them on agar plates at 37 ℃ for 24 h.

### Demonstration of antibacterial mechanisms of OEOs

#### Acid production

Acid production of *S. mutans* was evaluated by a glycolytic pH drop assay [17]. Briefly, the samples with different concentrations were added to bacterial suspension which was containing 1% glucose. After 24 h of cultivation, the pH of the cultures was determined using a pH meter (SevenCompact, Mettler Toledo). Before inoculation, the initial pH of the medium with samples was also determined.

#### Hydrophobicity test

The hydrophobicity of *S. mutans* was measured based on microbial adhesion to hydrocarbon [18]. 3 mL of 10^5^ CFU/mL *S. mutans* suspension containing 1% sucrose mixed with different concentration of sample before being incubated for 24 h. Then bacteria were recovered at 3000 r/min for 20 min, washed thrice and dispensed with PUM buffer (1.8 g Urea, 7.26 g KH_2_PO_4_, 22.2 g K_2_HPO_4_‧3H_2_O, 0.2 g MgSO_4_, pH 7.1) at an λ_660_ between 0.5 and 0.6 (recorded as OD_1_). The tube was vigorously mixed for 60 s after 400 μL hexadecane was added to each 3 mL of resuspended bacteria. The mixtures were incubated at 37 ℃ for 15 min until the full separation of the two phases. The upper layer of the mixed solution was the oil phase, and the lower layer was the aqueous phase. The absorbance of the lower aqueous phase was measured at λ_660_ (recorded as OD_2_). The degree of hydrophobicity was determined by the following equation:

$$\mathrm{Hydrophobicity }\left(\mathrm{\%}\right)=\left[1-\frac{{\mathrm{OD}}_{2}}{{\mathrm{OD}}_{1}}\right]\times 100$$

#### Biofilm formation assay

Biofilm formation of *S. mutans* was visualized by staining with safranin [17]. Different concentrations of samples were incubated with *S. mutans* in BHI medium containing 0.1% sucrose in 35 mm polystyrene Petri dishes. After 24 h, all supernatants were removed and the dishes were rinsed with distilled water and dried. Then the biofilm was fixed with 4% paraformaldehyde and washed twice with distilled water. Biofilm formation was stained with 0.5% safranin and photographed.

#### Quantification of biofilm formation

Biofilm formation of *S. mutans* was quantified by crystal violet staining method [19]. Various concentrations of samples were incubated with *S. mutans* in 96-well plate. After 24 h incubation, discarding the supernatants and washing twice with sterile water, the biofilm in the wells were fixed with 4% paraformaldehyde and then rinsed with sterile water. Next, 100 μL of 0.4% crystal violet was added to each well and stained for 15 min. The staining solution was then taken from each well and cleaned with distilled water until the color was not decolorized before 95% ethanol was added to dissolve the crystal violet. At 540 nm, the isolated dye was quantified and calculated according to the equation:

$$\mathrm{Inhibitory rate }\left(\mathrm{\%}\right)= \left[1-\frac{\mathrm{S}}{\mathrm{C}}\right]\times 100$$

C and S were defined as the average absorbance of control and sample groups respectively.

#### Quantitative real-time polymerase chain reaction (PCR) analysis

Quantitative real-time polymerase chain reaction (qRT-PCR) was used to detect the mRNA level of OEOs on gene expression of *S. mutans* (Supplementary Table 1) [17]. 1/2 MIC concentration of the samples were treated. After 24 h of culture, total RNA was isolated from bacteria using a TRIzol reagent (Trans) and cDNA was synthesized by ReverTra Ace qPCR RT kit (TOYOBO). Before total RNA was extracted from samples, samples were fully grinded separately under liquid nitrogen. The amplification was performed using a CFX96™ real-time PCR system with QPCR SYBR Green Mixes (TOYOBO). 16S rRNA was used as an internal control. The mRNA level was calculated using the 2-ddCt method.

### Cytotoxicity assay

Immortalized human keratinocytes (HaCaT) cells were provided by Peking union cell resource center (Beijing, China). The HaCaT cells were cultured in DMEM supplemented with 10% FBS, 1% P/S and 1% glutamine and maintained in a humidified 5% CO_2_ incubator at 37 °C. The cytotoxic activities of samples were measured by colorimetric MTT assay [20]. The cells were seeded into a 96-well plate at a density of 2 × 10^5^ cells/mL. After 24 h incubation, different concentrations (0.005, 0.01, 0.05, 0.1, 0.5, 1 and 10 μL/mL) of sample were added to each well and cultured for 12 h. Then the supernatant was carefully removed and added to 10 µL of 5 mg/mL MTT. After being incubated at 37 °C for 6 h, the resulting formazan crystals were dissolved in 100 µL of sodium dodecyl sulfate solution (Macklin, Shanghai, China). The absorbance was measured using the microplate reader infinite M200PRO (Tecan) at the wavelength of 570 nm. The calculation of cell cytotoxicity was based on the MTT absorption percentage as follows:

$$\mathrm{Cell viability }\left(\mathrm{\%}\right)= \left[\frac{{\mathrm{OD}}_{\mathrm{sample}}-{\mathrm{OD}}_{\mathrm{blank}}}{{\mathrm{OD}}_{\mathrm{control}}-{\mathrm{OD}}_{\mathrm{blank}}}\right]\times 100$$

### Molecular docking

Molecular docking was performed using AutoDock Vina [21]. The structure of protein targets (SpaP, PDB ID: 3OPU) was downloaded from the PDB database (https://www.rcsb.org) or modeled by the protein prediction servers (SWISS-MODEL). Ligands were energy minimized by using MMFF94 and prepared by using openbabel GUI. Receptor proteins were energy minimized and prepared for docking by SWISS PDB viewer and AutoDock Tools software (ADT4) respectively. On the protein surfaces, the active binding pockets were predicted based on computational geometry theories using CASTp server (http://sts.bioe.uic.edu/castp/).

### Statistical analysis

Data were expressed as mean ± standard deviation of at least triplicate experiments. Statistical tests were performed using the Micorcal™ Origin 2019b (Northampton, MA, USA). The parameters were analysed by ANOVA (one-way classifications) followed by Duncan Post hoc multiple comparisons.

## Results

### Chemical composition of the OEOs

Knowing the chemical composition of essential oils can lead to a better understanding of their biological properties. The chemical composition of OEOs via GC–MS analysis resulted in the identification of 21 chemical components in OEO1 and 39 in OEO2 (Table 1). In terms of bioactive components, monoterpenes and sesquiterpenes dominated. There were 18 identical components in these two essential oils. Carvacrol, belonging to oxygenated monoterpene, was the main ingredient and comprised 57.96% of the OEO1 and 59.40% of the OEO2. In addition, several other natural compounds were reported, including *p*-cymene (17.32% in OEO1, 10.37% in OEO2), *γ*-terpinene (10.67% in OEO1, 8.74% in OEO2) and *β-*myrcene (2.21% in OEO1, 2.56% in OEO2). Hence, two different samples used for this experiment were carvacrol-rich oregano essential oils with different geographical distributions.

**Table 1:** The main components of the OEOs

Compounds	RI	Content (Peak area%)	
		**OEO1**	**OEO2**
**Monoterpene hydrocarbons**		**35.32**	**26.97**
*α*-pinene	1028	1.50	0.97
*α*-thujene	1028	0.79	0.72
*α*-fenchene	1060	0.08	-
camphene	1071	0.46	0.31
*β*-pinene	1112	0.57	0.18
3-carene	1147	-	0.11
*β*-myrcene	1161	2.21	2.56
*α*-terpinene	1180	1.61	1.79
limonene	1200	0.11	0.35
*β*-phellandrene	1211	-	0.36
*β*-cis-ocimene	1235	-	0.30
*γ*-terpinene	1246	10.67	8.74
*p*-cymene	1272	17.32	10.37
terpinolene	1283	-	0.21
**Sesquiterpene hydrocarbons**		**1.96**	**5.54**
copaene	1492	-	0.07
*β*-bourbonene	1526	-	0.05
caryophyllene	1595	1.87	2.62
cis-*β*-farnesene	1662	-	0.10
humulene	1667	0.05	0.33
*γ*-muurolene	1692	-	0.14
( +)-ledene	1697	-	0.07
*β*-bisabolene	1727	0.04	1.62
*δ*-cadinene	1758	-	0.44
*γ*-cadinene	1765	-	0.10
**Oxygenated monoterpenes**		**61.72**	**64.37**
eucalyptol	1213	0.20	-
trans-sabinene hydroxide	1466	-	0.40
linalool	1547	3.45	0.72
trans-*β*-terpineol	1567	-	0.11
bornyl acetate	1581	-	0.09
( +)-3-thujanol	1635	-	0.24
(-)-borneol	1675	0.06	-
*α*-terpineol	1697	-	0.17
endo-borneol	1702	-	0.61
geraniol	1847	-	0.06
carvacryl acetate	1882	-	0.11
thymol	2189	0.05	2.46
carvacrol	2236	57.96	59.40
**Oxygenated sesquiterpenes**		**0.45**	**0.22**
Caryophyllene oxide	1989	0.45	0.22
**Others**		**0.52**	**2.71**
3-octanone	1253	-	0.16
1-Octen-3-ol	1450	0.16	0.62
Carvacrol methyl	1601	0.36	1.88
ether			
*p*-Cymen-8-ol	1852	-	0.05
**Total identified**		**99.97**	**99.81**

The samples were essential oils of *Origanum vulgare* L*.* (OEO1, origin: Bosnia) and *Origanum heracleoticum* L*.* (OEO2, origin: France)

### In vitro antibacterial activity

**Fig. 1:**
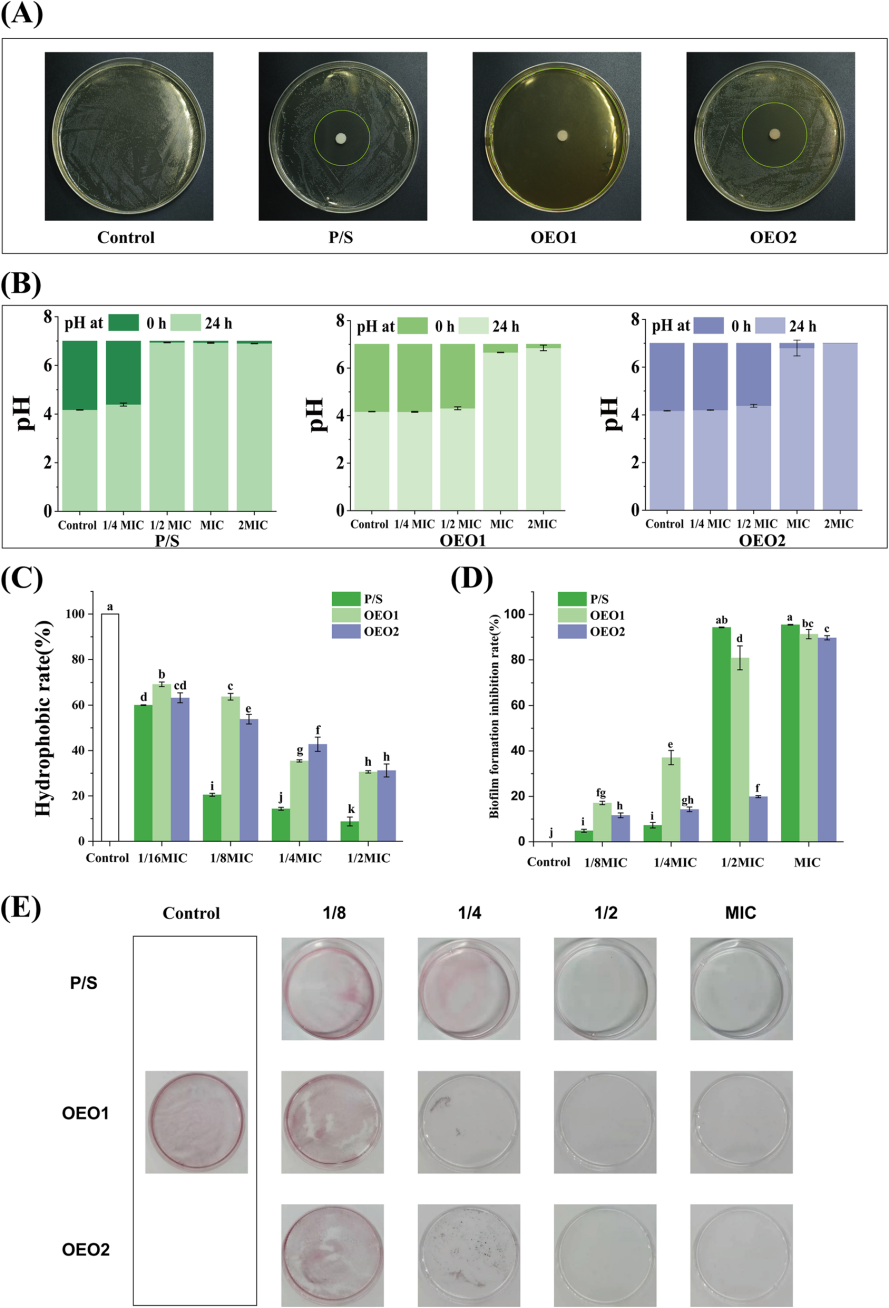
Antibacterial activity of OEOs. Parallel reactions to which no drug was added were used as controls. **A** Antibacterial activities of OEOs. **B** Effect of OEOs on acid production. The pH values of the all samples were all 7.01 as indicated by the dark columns, which represented the pH values of each sample prior to inoculation (0 h). Following inoculation and a 24-h culture period, the pH values of each sample were represented by the light-colored columns. **C** Effect of OEOs with various concentrations on hydrophobicity of *S. mutans*. **D** Inhibitory effect of OEOs on biofilm formation of *S. mutans*. The biofilm mass can be determined by dissolving crystal violet attached to the biofilm. **E** Effect of OEOs on biofilm formation on polystyrene dishes by *S. mutans.* The red-stained parts represented the deposited biofilm. Equal letters represent no differences while different ones represent significant differences (*p* < 0.05)

The antibacterial activity of different OEOs against the growth of *S. mutans* were measured by diameter of the inhibition zone (DIZ), MIC and MBC (Fig. 1A; Table 2). The DIZ can be used to preliminarily identify the sensitivity of microorganisms to samples. Based on preliminary screening of DIZ results, OEO1 and OEO2 effectively inhibited pathogen growth to distinct degrees. Compared with the positive control P/S, OEOs exhibited stronger antibacterial activity with larger halos (*p* < 0.05). By measuring the MIC, the minimum concentration of the sample required to inhibit the visible in vitro growth of *S. mutans* can be determined, which provides a basis for the selection of subsequent experimental concentrations. MBC identified the lowest concentration of sample that results in death of the microorganism. The MIC and MBC of OEOs were similar. The concentrations for MIC and MBC determination of OEO1 were 0.625 μL/mL and 2.5 μL/mL. The OEO2 had the MIC of 0.625 μL/mL and MBC of 1.25 μL/mL.

**Table 2:** Antibacterial activities of the OEOs against *S. mutans*

Samples	DIZ * (mm)	MIC (μL/mL)	MBC (μL/mL)
OEO1	≥ 80^a^	0.625	2.5
OEO2	39.67 ± 0.81^b^	0.625	1.25
P/S	34.13 ± 0.85^c^	0.78125	6.25

^*^ Values represent means of three independent replicates ± SD. Different letters within a column indicate statistically significant differences (*p* < 0.05) for DIZ

### Inhibition of acid production

Acid production is one of the key cariogenic virulence of *S. mutans*. *S. mutans* can produce lactic acid that corroded tooth enamel, so it was necessary to inhibit its ability to produce acid. To investigate whether OEOs can inhibit the production of organic acid from glycolysis in *S. mutans*, the change in pH was measured (Fig. 1B). If, after 24 h, the pH value of the sample was consistent with that at 0 h, it means that the sample at this concentration can inhibit the acid production ability of *S. mutans*. The initial pH of the negative control before culture was 7.01, while the pH declined to 4.17 after culture. After 24 h of inoculation, the pH of OEO1 and OEO2 at concentrations less than or equal to 1/2 MIC were close to the control group, indicating that the drop in pH of the solution caused by acid production by *S. mutans* could not be inhibited. Under the MIC concentration, OEO1 and OEO2 can inhibit the organic acid production by *S. mutans*. As a positive control, P/S inhibited acid production at the concentration of 1/2MIC, resulting in a pH of 6.94.

### Cell surface hydrophobicity

The hydrophobicity of cell surfaces was closely linked to the formation of biofilms, as hydrophobic cells are more likely to adhere to hydrophobic surfaces. And bacterial adhesion was the first step in biofilm formation. In high-caries patients, hydrophobic bacteria were more prevalent than in low-caries patients. Therefore, we determined whether OEOs inhibited the hydrophobic properties of *S. mutans* through microbial adhesion to hydrocarbons (MATH) assay, which was assessed by the hydrophobic interaction of n-hexadecane and *S. mutans* (Fig. 1C). The lower the hydrophobicity, the weaker the adhesion ability of bacteria. In comparison to control, 1/16 MIC of OEOs can significantly inhibit hydrophobicity of *S. mutans* (*p* < 0.05).

### Inhibitory effect of OEOs on biofilm formation

*S. mutans* developed virulence through the formation of biofilms. Biofilms were highly structured microbial communities that protected embedded bacteria and facilitated the creation of highly acidic microenvironments. The results revealed that the concentrations of 1/2–1 MIC of OEOs almost totally inhibited biofilm formation (Fig. 1D-E). The 1/2 MIC concentration of OEO1 showed 80.89% inhibition effect and the MIC concentration of OEO2 inhibited 89.71% of the biofilms.

### Inhibitory effect of OEOs on expression of virulence factor

**Fig. 2:**
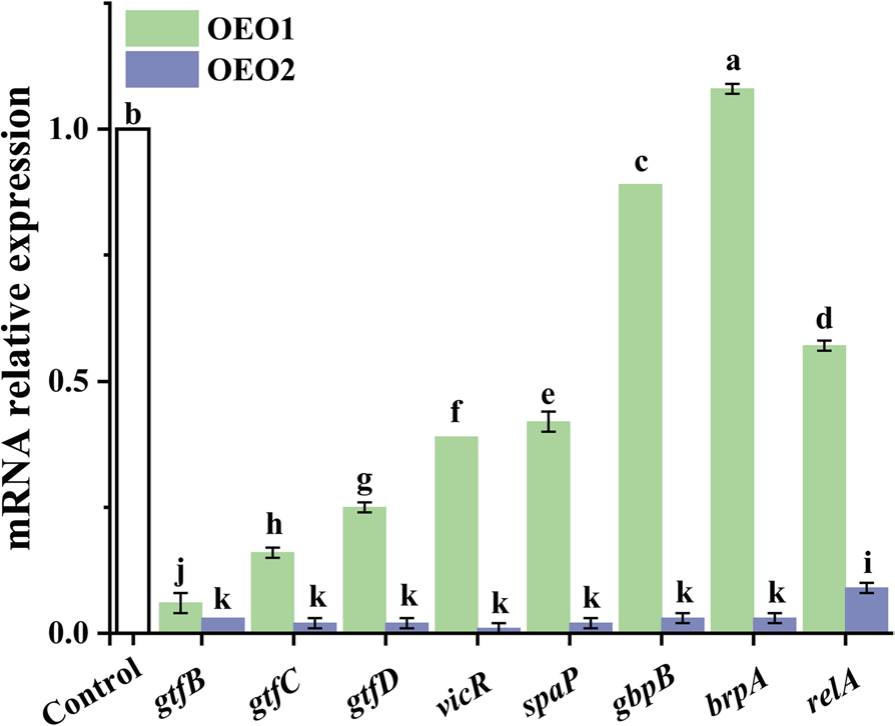
Effect of 1/2MIC concentration of OEOs on gene expression of key virulence of *S. mutans*. Equal letters represent no differences while different ones represent significant differences (*p* < 0.05)

To gain insight into gene expression, real-time PCR analysis was used to quantify the effect of OEOs at 1/2 MIC on *S. mutans* (Fig. 2). Among the eight studied genes, five genes were found to be involved in extracellular polysaccharide synthesis and biofilm formation (*gtf*B, *gtf*C, *gtf*D, *spaP* and *gbp*B). Genes *vic*R, *rel*A and *brp*A are primarily responsible for the cell’s adaptive response to environmental stress conditions. In general, all tested genes were almost obviously downregulated by OEOs, except for OEO1-treated *brpA* gene. In contrast, OEO2 has a stronger effect than OEO1.

### Molecular docking

**Fig. 3:**
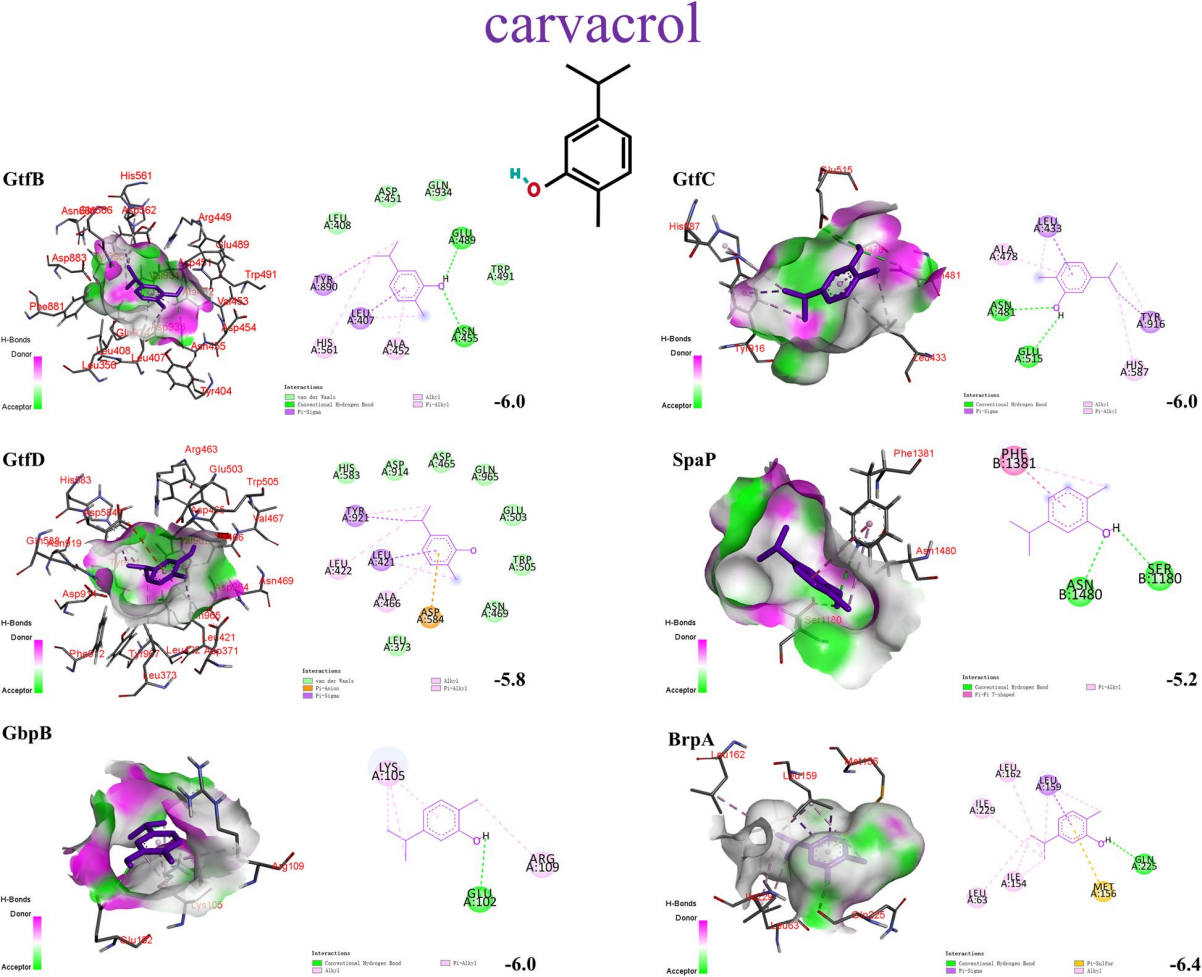
Three and two-dimensional predicted interaction view of carvacrol and varies virulence factor proteins residues. Red and green amino acids represented the H-bond donor and acceptor, respectively

**Fig. 4:**
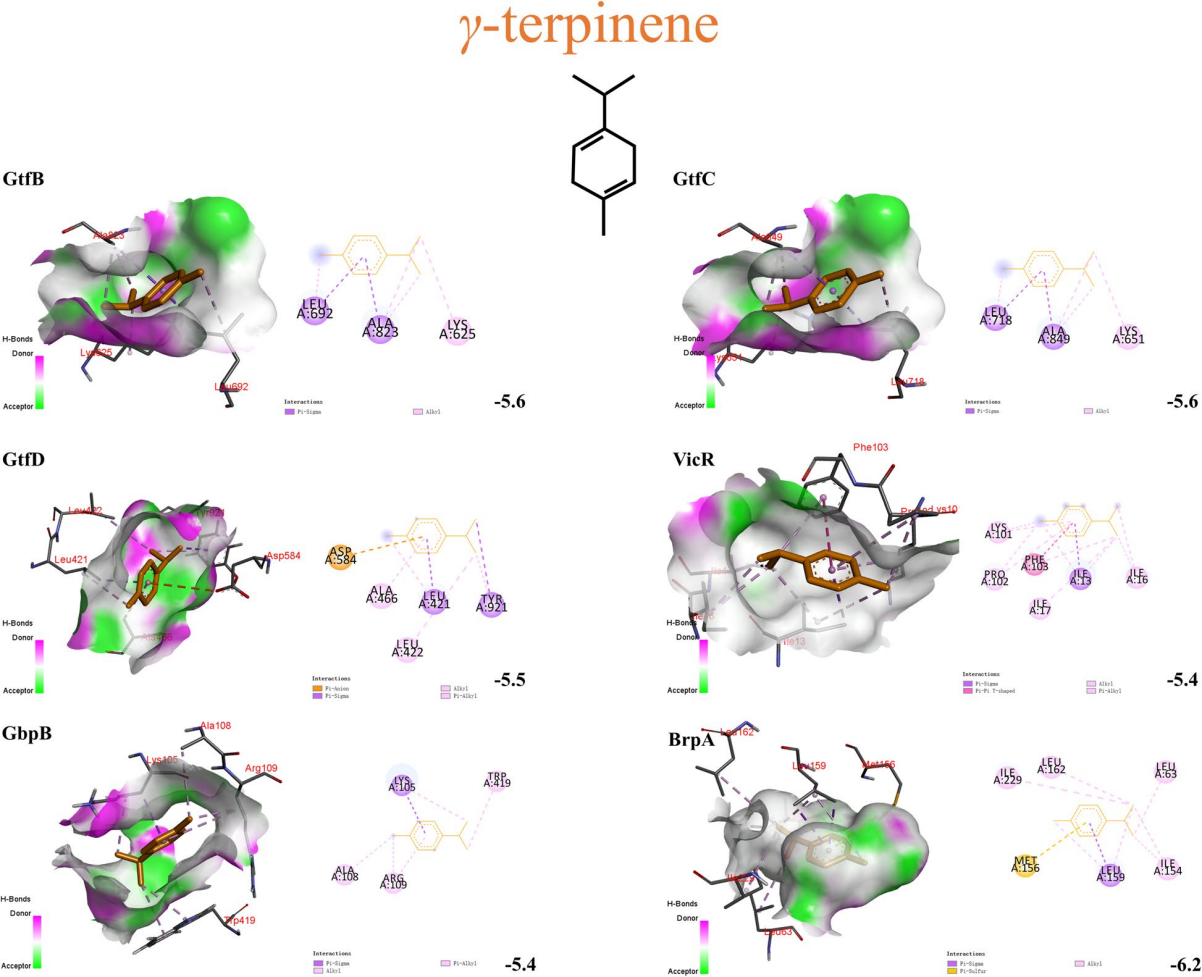
Three and two-dimensional predicted interaction view of *γ*-terpinene and varies virulence factor proteins residues. Red and green amino acids represented the H-bond donor and acceptor, respectively

**Fig. 5:**
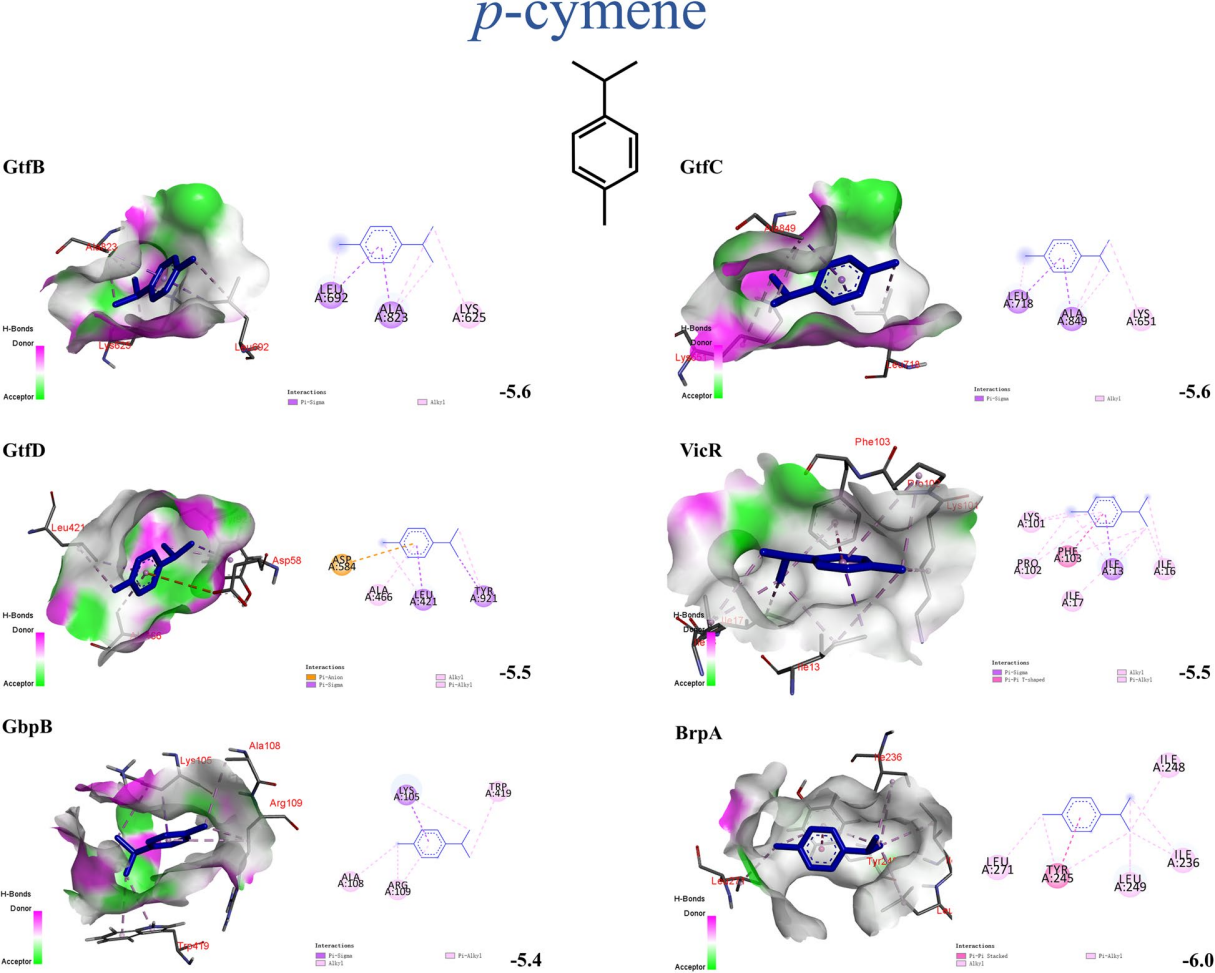
Three and two-dimensional predicted interaction view of *p*-cymene and varies virulence factor proteins residues. Red and green amino acids represented the H-bond donor and acceptor, respectively

There are many factors that affect the chemical composition and content of essential oils, including plant type or variety, geographical location of plants, climate, processing methods, etc. [13]. Therefore, by clarifying the interaction between monomers and proteins in the whole process, we can better analyze its mechanism of action and conduct quality control management of essential oils. The homology modelling was done for seven virulence factors and the 3D structural evaluation were shown in Supplementary Table 2. The predicted model all showed very few local errors in the amino acid sequence, and the chain conformation was suitable for docking. The molecular docking results showed that carvacrol had good affinity to GtfB (-6.0 kcal/mol), GtfC (-6.0 kcal/mol), GtfD (-5.8 kcal/mol), BrpA (-6.4 kcal/mol), SpaP (-5.2 kcal/mol) and GbpB (-6.0 kcal/mol). *γ*-terpinene and *p*-cymene bound to the pockets of the upstream gene protein VicR and downstream gene GTF, which were responsible for regulating exopolysaccharide formation, in the favorable docked pose (lower binding energy) Furthermore, both of them bound tightly to GbpB and BrpA. Only the RelA, to which all three compounds bound poorly. There were several interactions exist in the aforementioned binding, including pi interactions (pi-pi, Anion-pi, Sigma-pi, Alkyl-pi, Amide-pi, Sulfur-pi), van der Waals interactions, alkyl and hydrogen bond between ligands and receptor proteins. Notably, the chemical structures of these three ligands have certain similarities. But they may bind in different orientations or sites. Carvacrol can generate more strong hydrogen bonds with receptor proteins because of its phenolic hydroxyl group. Further details of good docking results such as three-dimensional binding pose, binding cavity, and two-dimensional interaction, binding score of predicted binding sites were listed in Figs. 3, 4, 5 and Table 3. The results of remaining molecular docking are listed in Supplementary Fig. 1.

**Table 3:** Molecular docking results of target and ligands

**Ligands**			**Docking score (Kcal/mol)**						
	**GtfB**	**GtfC**		**GtfD**	**VicR**	**SpaP**	**GbpB**	**BrpA**	**RelA**
carvacrol	-6.0	-6.0		-5.8	-4.9	-5.2	-6.0	-6.4	-4.8
*γ*-terpinene	-5.6	-5.6		-5.5	-5.4	-4.7	-5.4	-6.2	-4.3
*p*-cymene	-5.6	-5.6		-5.5	-5.5	-4.7	-5.4	-6.0	-4.3

### Cytotoxic effects of OEOs

**Fig. 6:**
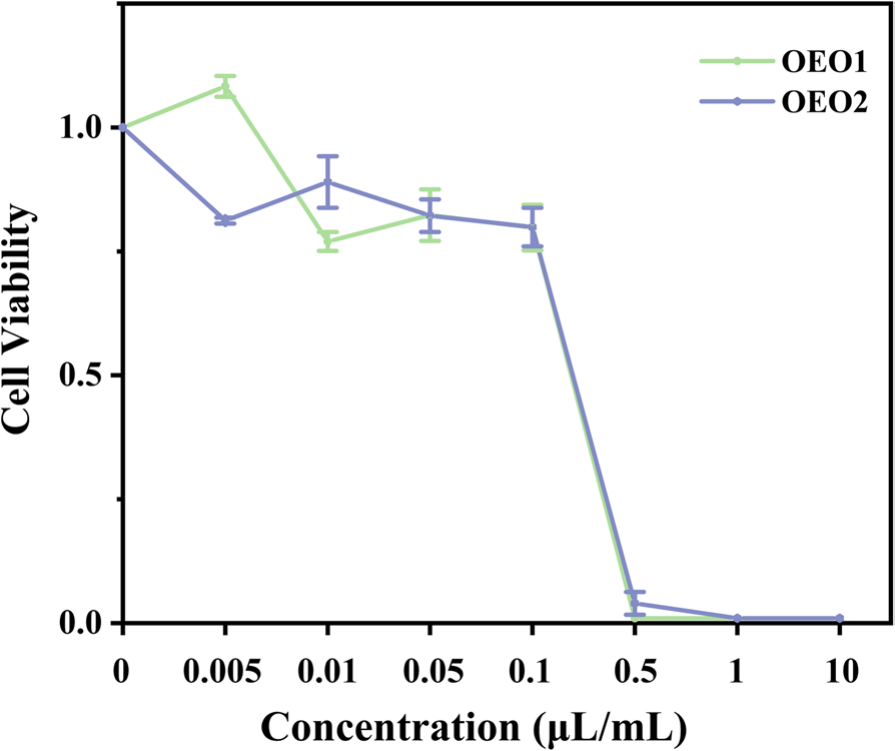
Cytotoxicity of OEOs in HaCaT cells

The cytotoxicity test was a biological evaluation test that used tissue cells to observe the effect of samples on cell growth in vitro. The aim of this experiment was to initially evaluate the effect of OEOs on the viability and growth of HaCaT cells. When the concentration was less than 0.1 μL/mL, OEOs demonstrated no negative effects on cell viability and growth (Fig. 6). At this concentration, OEOs had an inhibitory effect on the growth and virulence of *S. mutans*.

## Discussion

*Origanum vulgare* is a widespread and highly variable species. Oregano is either predominated by a cymyl-, sabinyl-, or linalool-chemotype. Subspecies rich in essential oils (2% or more) commonly accumulate large quantities of phenolic monoterpenes deriving from the “cymyl”-pathway (mainly carvacrol and/or thymol and their biosynthetic precursors *γ*-terpinene, and *p*-cymene [22]. The main constituents of the two different OEOs used in this study are carvacrol, *p*-cymene, *γ*-terpinene and thymol, consistent with the results of previous studies [23, 24].

**Fig. 7:**
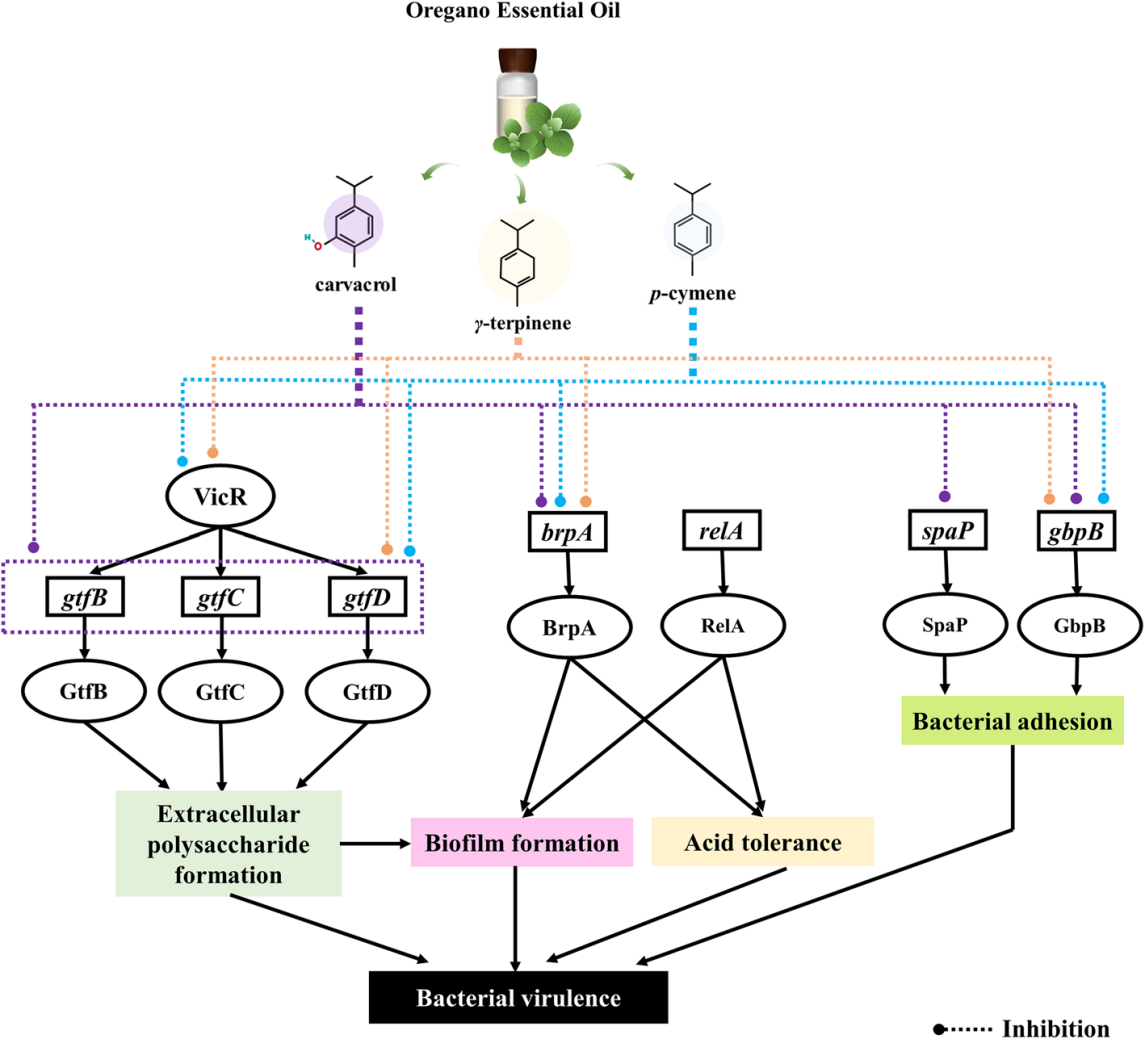
The mechanism underlying the anti-cariogenic effect of oregano essential oils against *S. mutans*

As shown in this study, the essential oils of *Origanum vulgare* L. and *Origanum heracleoticum* L. exhibited a satisfactory anti-microbial effect. Their MIC and MBC concentrations were similar. Dental plaque biofilm is the critical factor causing dental caries. There are three stages of its formation. Formation of an acquired pellicle, initial adhesion, and community maturation. MIC and sub-MIC concentrations of the OEOs nearly completely inhibited biofilm formation of *S. mutans*. This was not least due to the OEOs significantly reducing the hydrophobicity of *S. mutans* (*p* < 0.05), thereby decreasing the success of its implantation and adhesion and rendering it more susceptible to detachment from tooth surfaces [25]. This was also confirmed by the formation of two conventional hydrogen-bonding interactions between the hydroxyl groups of carvacrol in OEOs and SER-1180 and ASN-1480 of SpaP. Moreover, OEOs can regulate its downstream genes *gtfB*/*C*/*D* by inhibiting *vicR*, preventing *S. mutans* from metabolizing dietary sucrose into glucans (Fig. 7). These components provide a basic scaffold for biofilm development, promoting local microbial cells accumulation within a diffusion-limiting polymeric matrix that protects the embedded bacteria and reduces the antibiotic concentrations. Regarding the docking results, carvacrol, *γ*-terpinene and *p*-cymene also had direct interaction with GtfB/C/D and surface-associated glucan binding proteins GbpB. These results exemplified the additive effect of carvacrol, *γ*-terpinene and *p*-cymene, and further indicated that the MIC and sub-MIC concentration of OEOs can disrupt the sucrose-independent and sucrose -dependent adhesion of *S. mutans*, to reach a good caries-preventive effect.

In order to occupy the entire territory in dental plaque, *S. mutans* can rapidly acidify the microenvironment and kill competitors [26]. Prolonged periods of plaque acidification allow for the demineralization of the tooth enamel and thereby caries. But it can thrive in a low pH environment via initiating an acid tolerance response to enhance the plasma membrane barrier function and induce the alkalization of the cytoplasm [27]. Throughout these processes, the cell envelope protects the cell from the environment, maintains cell shape, acts as a molecular sieve. Biofilm regulatory protein A (BrpA) plays a vital role. Previous studies have shown that the absence of BrpA in *S. mutans* leads to increase autolysis and decrease viability, suggesting a role for BrpA in cell envelope integrity [28]. In this study, OEO2 effectively suppressed *brpA* expression (*p* < 0.05). Carvacrol and γ-terpinene were tightly wrapped by the BrpA and produced a favorable pi-sulfur noncovalent interaction. While the results also showed that OEO1 stimulation rather increased *brpA* expression (*p* < 0.05). The OEO1 may have disrupted the cell envelope of *S. mutans* to some degree and consequently increased transcription. We inferred other components of OEOs predominantly mediated inhibitory effects in *brpA*. Considering the inhibition capacity of OEO2 with 39 components was stronger than that of OEO1 with 21 components (*p* < 0.05), we can get into a conclusion that some trace components in OEO2 are supposed to play an important role in the virulence inhibition of *S. mutans*.

RelA mediates a regulatory mechanism known as the stringent response. Under the amino acid starvation, RelA alters expression of stress genes and inhibits an increase in amino acid biosynthesis, stable RNA, DNA, and cell wall synthesis [29]. Therefore, *S. mutans* exposed to 1/2 MIC OEO1 and OEO2 will adjust metabolism by favoring transcription of genes involved in stress tolerance at the expense of those essential for growth. The molecular docking scores showed that carvacrol, *γ*-terpinene and *p*-cymene were not optimal substrates for protein RelA. Similarly, other components of OEOs predominantly mediated inhibitory effects in *relA*.

From the above results, compared with antibiotics, which act in the single-molecule mode, the synergistic effect of the various components in oregano essential oil may be able to greatly reduce microbial resistance. This is mainly reflected in the fact that different components can interact with different binding sites and virulence proteins according to their own affinities.

## Conclusion

The present study showed that sub-MIC concentration of OEOs interfered with *S. mutans* growth and successfully inhibited its cariogenic virulence. Network pharmacology-based analysis identified potential antibacterial key compounds and their synergistic effects in complex OEO profiles. Due to the potent and diverse biological activities of OEOs, they may be a promising lead hit for the development of caries-preventive measures applied in further clinical applications. However, whether components such as carvacrol, *γ*-terpinene and *p*-cymene from OEO participate in the regulation should be investigated further, and the mechanism should be elucidated. In the future, the antimicrobial properties of OEOs and their active ingredients in multiple infection models, establishing the safety of optimal doses and continuing to examine their efficacy against bacterial strains isolated from patients are worth exploring.

## Supplementary Information

**Additional file 1:** **Supplementary Table 1.** Primer sequences used in qRT-PCR. **Supplementary Table 2.** Evaluation results of proteins 3D structures. **Supplementary Figure 1.** Three and two-dimensional predicted interaction view of three components and varies virulence factor proteins residues. Red and green amino acids represented the H-bond donor and acceptor, respectively.

## Data Availability

The data used to support the findings of this study are available from the corresponding author upon request.

## Abbreviations

*S. mutans*: *Streptococcus mutans*
OEO: Oregano essential oil
MIC: Minimum inhibitory concentration
MBC: Minimum bactericidal concentration
BHI: Brain heart infusion
P/S: Penicillin /streptomycin
HaCaT: Immortalized human keratinocytes cell
DMEM: Dulbecco's modified eagle medium
FBS: Fetal bovine serum;
GC‒MS: Gas chromatography-coupled to mass spectrometry
