# Supplementary material for: Antibacterial activity of oregano essential oils against Streptococcus mutans in vitro and analysis of active components
Source: BMC Complement Med Ther. 2023 Feb 21;23:61. doi: 10.1186/s12906-023-03890-4 (PMC9942419; doi:10.1186/s12906-023-03890-4)
Supplement: Supplementary file 1 — Additional file 1: Supplementary Table 1. Primer sequences used in qRT-PCR. Supplementary Table 2. Evaluation results of proteins 3D structures. Supplementary Figure 1. Three and two-dimensional predicted interaction view of three components and varies virulence factor proteins residues. Red and green amino acids represented the H-bond donor and acceptor, respectively. [file 12906_2023_3890_MOESM1_ESM.docx]

**Supplementary Table 1** Primer sequences used in qRT-PCR.

| Genes* | Gene description |  | Primer sequence (5’……3’) |
| --- | --- | --- | --- |
| *gtfB* | Glucosyltransferase-I | Forward | AGCAATGCAGCCAATCTACAAAT |
|  |  | Reverse | ACGAACTTTGCCGTTATTGTCA |
| *gtfC* | Glucosyltransferase-SI | Forward | GGTTTAACGTCAAAATTAGCTGTATTAGC |
|  |  | Reverse | CTCAACCAACCGCCACTGTT |
| *gtfD* | Glucosyltransferase-S | Forward | ACAGCAGACAGCAGCCAAGA |
|  |  | Reverse | ACTGGGTTTGCTGCGTTTG |
| *brpA* | Biofilm regulatory protein A | Forward | GGAGGAGCTGCATCAGGATTC |
|  |  | Reverse | AACTCCAGCACATCCAGCAAG |
| *spaP* | Cell surface antigen SpaP | Forward | GACTTTGGTAATGGTTATGCATCAA |
|  |  | Reverse | TTTGTATCAGCCGGATCAAGTG |
| *gbpB* | Secreted antigen GbpB/SagA | Forward | ATGGCGGTTATGGACACGTT |
|  |  | Reverse | TTTGGCCACCTTGAACACCT |
| *relA* | GTP pyrophosphokinase | Forward | ACAAAAAGGGTATCGTCCGTACAT |
|  |  | Reverse | AATCACGCTTGGTATTGCTAATTG |
| *vicR* | Response regulator | Forward | TGACACGATTACAGCCTTTGATG |
|  |  | Reverse | CGTCTAGTTCTGGTAACATTAAGTCCAATA |
| 16S rRNA | 16S rRNA | Forward | CCTACGGGAGGCAGCAGTAG |
|  |  | Reverse | CAACAGAGCTTTACGATCCGAAA |

*Based on the NCBI *S. mutans* genome database.

**Supplementary Table 2** Evaluation results of proteins 3D structures**.**

| Protein name | Model | ERRAT | Verify 3D | Favored region | Allowed Region | Non-allowed region |
| --- | --- | --- | --- | --- | --- | --- |
| brpA | I-tasser | 89.93 | 81.28 | 75.9 | 22.4 | 0 |
|  | Swiss-Model | 95.02 | 100 | 91.8 | 8.2 | 0 |
| gbpB | I-tasser | 81.19 | 35.96 | 69.2 | 21 | 3.5 |
|  | Swiss-Model | 94.09 | 44.79 | 89.3 | 8.5 | 0.8 |
| gtfB | I-tasser | 76.75 | - | 61.7 | 29.9 | 1.8 |
|  | Swiss-Model | 90.97 | 94.79 | 88.9 | 10.7 | 0 |
| gtfC | I-tasser | 76.87 | - | 64.6 | 27.8 | 3.2 |
|  | Swiss-Model | 92.05 | 94.91 | 89.8 | 10 | 0 |
| gtfD | I-tasser | 74.29 | - | 71.7 | 26 | 3.3 |
|  | Swiss-Model | 84.76 | 82.57 | 85.4 | 13.0 | 0.5 |
| relA | I-tasser | 99.53 | 80.54 | 82.3 | 14.8 | 1.5 |
|  | Swiss-Model | 97.33 | 76.12 | 96.8 | 3.2 | 0 |
| vicR | I-tasser | 89.4273 | 77.02 | 77.7 | 19.4 | 1.4 |
|  | Swiss-Model | 93.2127 | 84.98 | 90.0 | 9.1 | 0.5 |

**
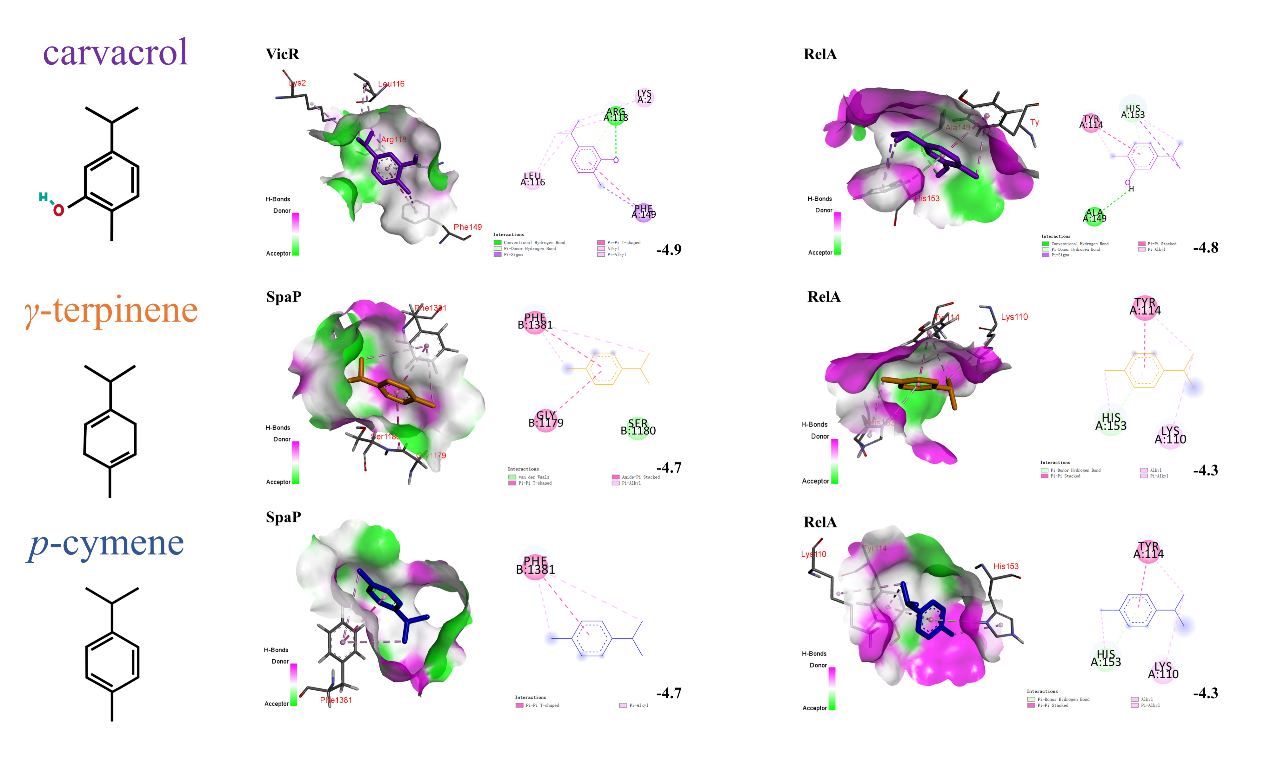
**

**Supplementary Figure 1** Three and two-dimensional predicted interaction view of three components and varies virulence factor proteins residues. Red and green amino acids represented the H-bond donor and acceptor, respectively.
